# Heart Affections

**Published:** 1858-07

**Authors:** 


### HEART AFFECTIONS.

THERE is not a pin's point surface of the external portion of the body which does not give instantaneous warning of the slightest invasion from without: it is our guard against external dangers. But the more important internal organs are so well protected by the casings which surround them, that they do not require such sensitiveness; hence it is not thrown away on them. For example: It is generally supposed, that if the heart is touched, we would instantly die. So far from this being true, bayonets and bullets have been driven into the heart of men and animals without causing death. The bullet which caused the death of WILLIAM POOLE was found imbedded in the solid substance of the heart, and yet he lived eleven days after he was shot.

The great HARVEY brought CHARLES II. to a British nobleman who had an opening in his chest, through which the heart could be seen and felt. The king touched it with his finger, but the nobleman was not aware of it, unless when he saw the fingers in the cavity. And yet, this same heart responds to every emotion of joy, or grief, or passion, or alarm.

Persons often trouble themselves uselessly, about having disease of the heart or lungs, because they have pain thereabouts. These are good signs, generally, as showing that the pains are external to these organs, for there can be no pain where there are no nerves. The fact is, the most certainly fatal affections of these organs give no note of warning by pain, until within the last brief hours of life. The very substance of the lungs and heart are often eaten through, eaten away, without a remote suspicion on the part of the patient that such was the case. The celebrated clerical orator, JOHN NEWLAND MAFFITT, died of a literally broken heart—not an emotional breaking, but a structural rupture; it had slowly decayed, rotted away, until so near through, that its functions could not be performed, when pains came on, and he died in a few hours; on examination, it was found that this destruction of parts had been going on for months, most probably. It was authoritatively stated, at the time of GENERAL JACKSON'S death, that one-third of one side of his lungs had been destroyed, apparently twenty years before. The practical inference is this, that pains about the lungs and heart, off and on, for weeks and months, generally indicate the absence of seriously critical diseases.

